# A novel concentrated growth factor (CGF) and bio-oss based strategy for second molar protection after impacted mandibular third molar extraction: a randomized controlled clinical study

**DOI:** 10.1186/s12903-023-03411-2

**Published:** 2023-10-12

**Authors:** Shoufu Sun, Xiaodong Xu, Zhongxiao Zhang, Ying Zhang, Wenjia Wei, Ke Guo, Yunan N. Jiang

**Affiliations:** 1 Department of Stomatology, Tongren Hospital, Shanghai Jiao Tong University School of Medicine, 1111 Xianxia Road, Shanghai, 200336 People’s Republic of China; 2 Tongren Hospital, Hongqiao International Institute of Medicine, Shanghai Jiao Tong University School of Medicine, Shanghai, 200336 People’s Republic of China

**Keywords:** Impacted third molar, Bone defect, Clinical protection, Bone repair, CGF;long-term

## Abstract

**Background:**

The extraction of impacted mandibular third molars might cause large bone defects in the distal area of second molars. A new strategy was innovatively employed here combining autologous bone, Bio-Oss, concentrated growth factors (CGF) gel and CGF membrane for bone repair, and the present study aimed at exploring safety as well as short- and long-term efficacy of this new protocol clinically.

**Materials and methods:**

A total of 66 participants were enrolled in this randomized single-blind clinical trial, and randomly allocated to control group (only blood clots), test A group (autogenous bone, Bio-Oss with barrier membrane) and test B group (autogenous bone, Bio-Oss, CGF gel with CGF membrane). The postoperative outcomes including PoSSe scale, periodontal probing depth (PD), degree of gingival recession and computed tomography measurements were assessed at 3rd, 6th, 12th month. A p-value < 0.05 was considered statistically significant.

**Results:**

In PoSSe scale, no significant difference was observed except a significant alleviation of early-stage pain perception in test B group (p < 0.05). Also, test B group exhibited better effect on periodontal healing and gingival recession reduction after 6 months (p < 0.05). Both two test groups showed more new bone formation than the control group (p < 0.05). It is noteworthy that the bone repair of test B group was significantly better than that of test A at 3rd and 6th month (p < 0.05), yet no difference was observed at 12th month (p > 0.05).

**Conclusion:**

Both two test groups could achieve stable long-term efficacy on bone defect repair. The use of CGF gel and CGF membrane could accelerate early-stage bone repair, alleviate short-term pain after surgery, reduce long-term probing depth and relieve economic cost for patients. This new bone repair protocol is worthy of promoting by clinicians.

**Trial registration:**

This study was registered with the identification number ChiCTR2300068466 on 20/02/2023 at Chinese Clinical Trial Registry. Also, it was ethically approved from the institutional ethics committee at the Tongren Hospital, Shanghai Jiao Tong University School of Medicine, Shanghai, China (No:2023-010-01), and has been conducted in accordance to the guidelines of the declaration of Helsinki. Written informed consent was obtained from all participants in the study.

**Supplementary Information:**

The online version contains supplementary material available at 10.1186/s12903-023-03411-2.

## Background

The mandibular third molar is a newly erupted tooth at young age, and located distally to the second molar. It is often impacted due to insufficient space or improper eruption direction. Frequent recurrence of the third molar pericoronitis might cause severe bone resorption in the distal area of second molar [1–3]. Large amount of bone defect after extraction of the horizontal impacted third molar has become a prominent potential risk factor for the second molar. Once ignored, the bone defect will cause oral health problems including gingival recession, pain from hot or cold stimulation, periodontal pocket formation, tooth loosening, and even loss of tooth, which might severely impair life quality of the patients [4, 5]. After third molar removal, if the socket is left to heal naturally, the bone height could fail to recover, and deep periodontal pocket will appear in the distal area of second molar. Worse still, infrabony pocket caused by alveolar bone loss or even postoperative periodontitis will occur. Impacted mandibular third molar is very common clinically, mainly at the age between 20 and 30 years old. How to alleviate the clinical symptoms while repair the bone defects caused by third molar extraction has become the most concerned problem of many surgeons recently. In previous studies, some scholars believed that flap incision design, periodontal curettage and guided tissue/bone regeneration (GTR/GBR) could improve the distal bone repair of mandibular second molar [6]. Among them, GBR had always been regarded as the best method for the treatment of bone tissue defects, which promoted new bone formation and restored the original anatomical structure and function [7]. In previous reports, some scholars employed bone substitutes, platelet rich fibrin (PRF) or autogenous demineralized dentin matrix (DDM) to preserve the alveolar sites of the extraction sockets, achieving certain clinical effects [6, 8]. Elgali et al. [9, 10] implanted coral composite artificial bone or hydroxyapatite together with other materials after the removal of impacted third molar, which showed good bone conductivity and bone induction, yet poor bone generation. Kim Y et al. [8] indicated that DDM could improve bone defect repair at the distal site of the mandibular second molar after third molar extraction. Autologous bone transplantation is the gold standard for bone repair, but its clinical application is restricted due to certain limitations, such as the need for a second operative area, complications in the donor area, the hospitalization or general anesthesia requirements and the limited bone supply.

Concentrate growth factors (CGF) has become one of the new methods for alveolar bone defect reconstruction because of its strong ability of bone regeneration. CGF is a new biomaterial discovered after platelet-rich plasma (PRP) and platelet rich fibrin (PRF), which contains a variety of high concentration growth factors and fibrin [11]. It can prominently improve the osteogenic quality and shorten the osteogenic time. Previous studies [12, 13] have shown that CGF contained almost all the growth factors in centrifugal blood, and its slow release was more similar to the natural process of tissue healing. CGF exhibited stronger promoting effect on soft and hard tissue healing compared with PRP and PRF. Previous reports demonstrated that CGF could effectively stimulate the proliferation of osteoblasts, increase the differentiation rate of osteoblasts by 4 ~ 6 times, and significantly improve the process of bone healing [14, 15]. Most evidence showed that CGF exhibited obvious advantages for early osteogenesis, yet some studies pointed out that there was no significant difference after 4 months [16, 17]. Xu et al. [18] also found that CGF could reduce periodontal depth of intrabony defects. When mixed with Bio-Oss, CGF showed better results in the early period, and the long-term effect was more stable than CGF used alone.

Based on comprehensive consideration, we innovatively proposed a new bone repair protocol: combining autogenous bone, Bio-Oss, CGF gel and CGF membrane which provided a new strategy for the clinical protection of the second molar. So far there was no long-term follow-up report of this new bone repair protocol. Therefore, detailed investigations on the efficacy were urgently needed before further clinical promotion.

## Materials and methods

### Study design

This study was prospective randomized, single-blind, controlled clinical trial, designed in accordance with guidelines outlined in the Consolidated Standards of Reporting Trials (CONSORT) statement. Participants were recruited from patients who underwent mandibular third molar extraction at our hospital from September 2020 to December 2021. This study was ethically approved by the institutional ethics committee at the Tongren Hospital, Shanghai Jiao Tong University School of Medicine, Shanghai, China (No:2020 − 152), and was conducted in accordance with the guidelines of the declaration of Helsinki. Written informed consent was obtained from all participants in the study.

### Inclusion and withdrawal criteria

Participants in this clinical trial must meet all the following criteria:

1) The subjects shall be over 18 years old (inclusive), regardless of gender; 2) The impacted mandibular third molars were showed mesioangular or horizontal, Pell &Gregory grade [19] (class II, Level B) on the panoramic X-ray, so the difficulty values were the same; 3) No periodontal disease, other acute infectious diseases, mental diseases, or systemic diseases; 4) Volunteers sign informed consent forms.

Withdrawal criteria: All subjects who sign the informed consent form and are eligible to enter the study are shedding cases no matter when they withdraw from the study for any reason, as long as they do not complete the observation period stipulated in the protocol. (1) The subjects voluntarily quit the test for various reasons; (2) Due to adverse events, especially serious adverse events, the ethics committee considers suspending the study from the ethical and moral perspective; (3) If the bone materials need to removed or other treatment is necessary because of the adverse events during the trial; (4) It is necessary for the researcher to stop the study from the medical point of view; (5) Those who need to stop the experiment for other reasons.

Before participating in the study, all subjects were informed of the purpose and operative process of the study and signed a tooth extraction informed consent and a study informed consent. The name, age, sex, operation note of the patients were recorded on the tooth extraction day.

### Sample size calculation

The sample size was calculated with PASS 15 software. When the power of test is 0.8, The minimum sample size required was 20 subjects per group. Considering the loss of follow-up rate of 20%, we collected at least 25 subjects in each group.

### Randomization

**Fig. 1:**
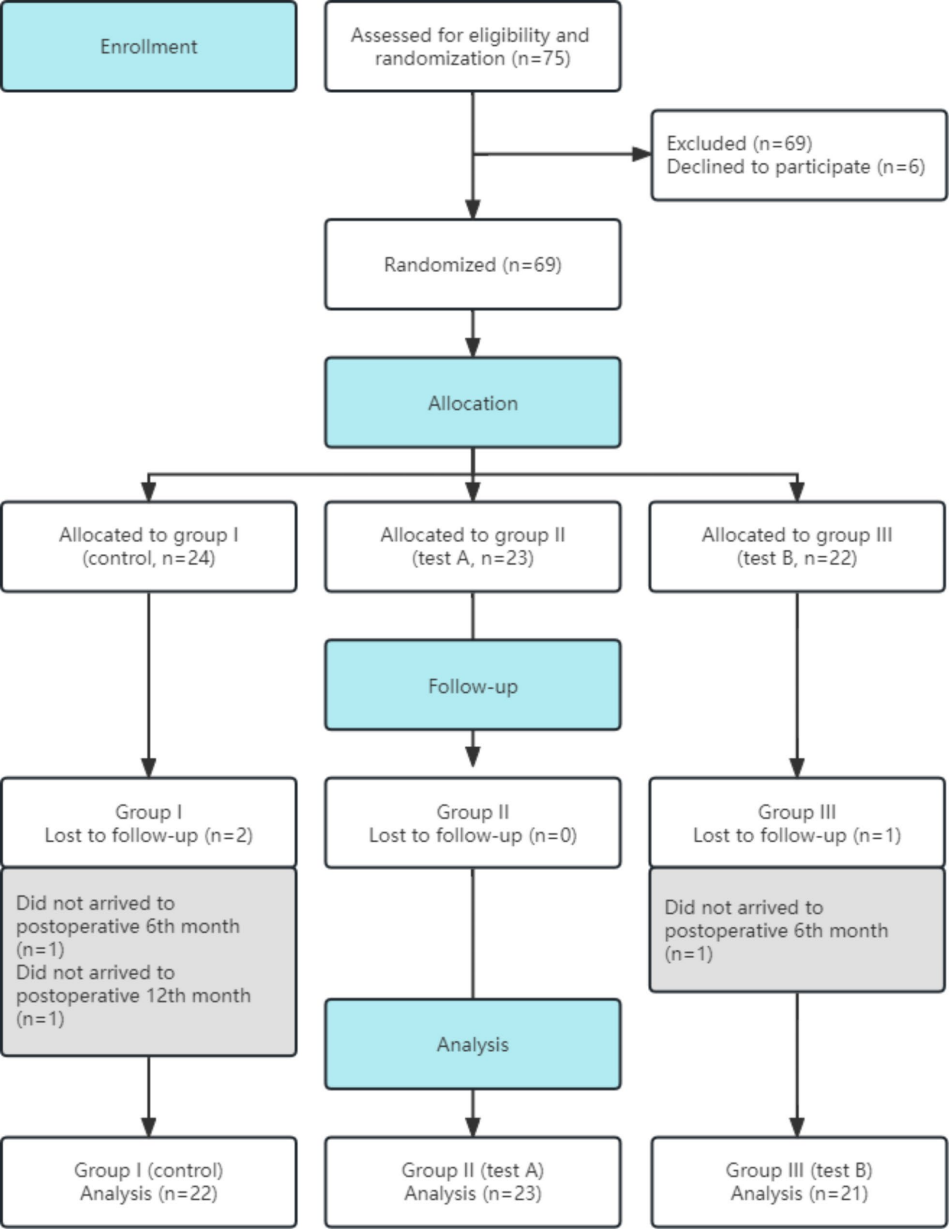
Flowchart of patient participation

The patients participating in the clinical trial in the stomatology department of our hospital were randomly divided into three groups, and the surgery was performed by the same experienced clinician. Three envelopes, labeled “Group I”, “Group II” and “Group III”, were prepared before surgery. The surgeon opened it after the patient made a choice. A total of 75 patients were collected, of whom 9 dropped out of the trial and 66 were enrolled (Fig. 1).

Group I (control): in which only blood clot, including 22 patients;

Group II (test A): in which autogenous bone, Bio-Oss and barrier membrane were applied in the extraction socket, including 23 patients;

Group III(test B): in which autogenous bone, Bio-Oss, CGF gel and CGF membrane were applied in the extraction socket, including 21 patients.

### Study variables

The primary variables of this clinical study were Bio-Oss and CGF application.

The primary outcome variables were the effect of bone defect repair, including the height and width of bone repair in the extraction socket, angle of the distal infrabony pocket of the second molar at 3rd month, 6th month and 12th month.

The secondary outcome variables were post operative symptom severity scale (PoSSe), probing depth (PD) and degree of gingival recession.

### Preparation of CGF

CGF was prepared 15 min before tooth extraction. Patients’ fresh venous blood (20 ml) was collected and placed in 2 sterile test tubes without anticoagulant. One tube was prepared for extraction cavity filling, and the other was utilized to prepare CGF membrane. The test tubes were placed in the centrifuge according to the following procedures: 30 s acceleration, 2 min×2700 rpm, 4 min×2,400 rpm, 4 min × 2,700 rpm, 3 min×3,000 rpm, 36 s deceleration, stop [20]. After taking out the test tube, the blood in the tube showed a three-layer structure, and the yellow jelly of middle layer was taken as CGF gel. The CGF gel was pressed lightly by a mold, and the liquid components could be extruded from it, then we can obtain the self-concentrated growth factor membrane.

### Surgical protocol

Patients in three groups received inferior alveolar nerve, lingual nerve, and buccal nerve block anesthesia with 2% lidocaine 5 mL plus 1:200,000 epinephrine. After successful anesthesia, the same clinician removed the impacted mandibular third molars by a standardized minimally invasive tooth extraction method, according to the impaction conditions.

**Fig. 2:**
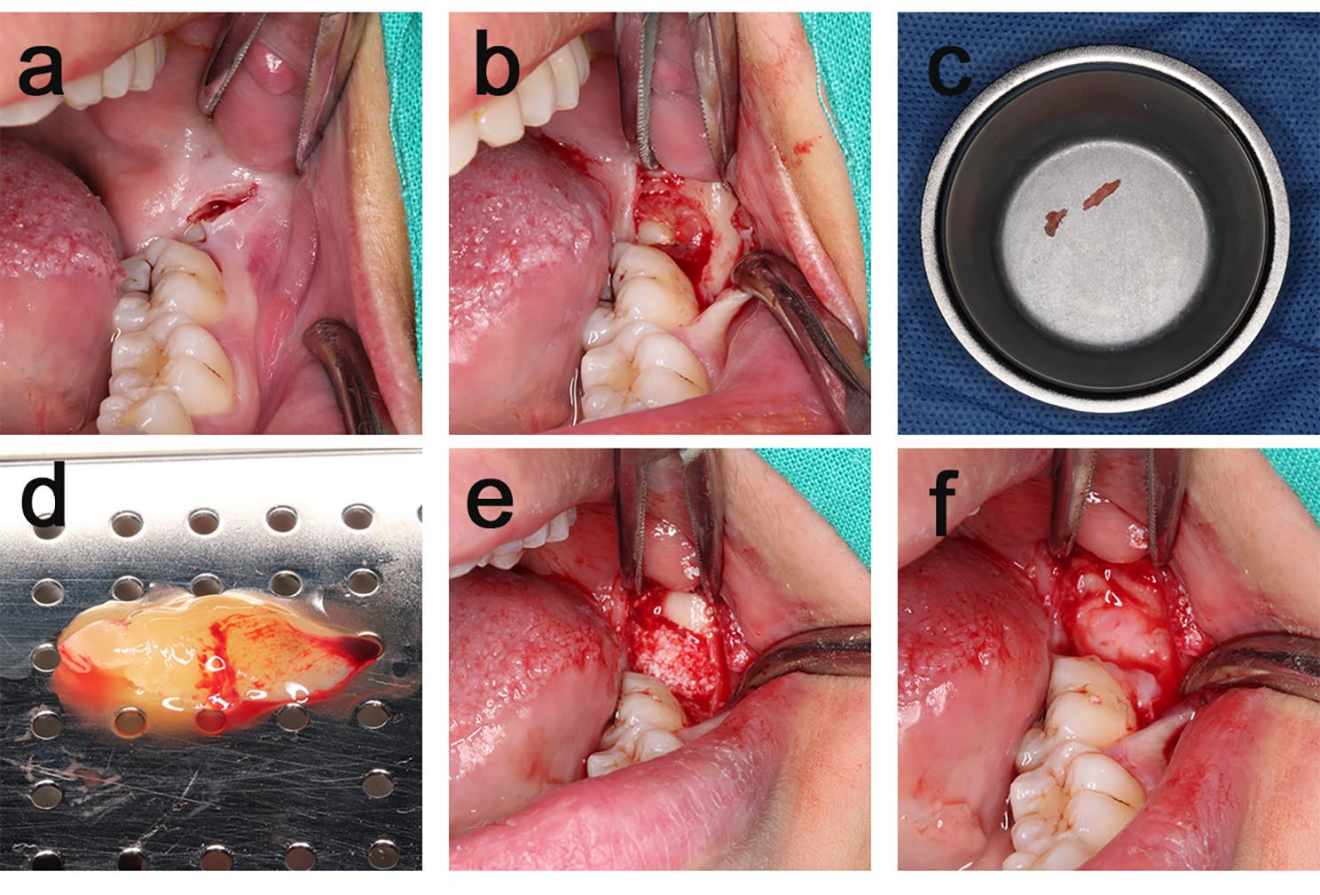
Surgical Procedure (**a**) 45°oblique incision, (**b**) remove bone by ultrasonic, (**c**) autogenous bone, (**d**) CGF preparation, (**e**) filing the socket, (**f**) CGF membrane was placed on the surface

Before surgery, patients rinsed their mouths with 0.12% clorhexidine gluconate as an antiseptic mouthwash for 60s. Iodophor (Lanso Skin Mucosa Disinfectant, 60ml, active ingredients and content: iodine, effective iodine content (w/v) 0.45-0.55%, chlorhexidine acetate content (w/v) 0.028-0.034%) was used for oral and maxillofacial disinfection. A 45°oblique incision was made on the distal buccal mucosa of the second molar with No. 11 blade, and the envelope flap was turned to the bone surface (Fig. 2 a). Ultrasonic bone knife was used to remove the resistant bone of the third molar (Fig. 2 b), and the removed autogenous bone would be used as bone filling material for the extraction socket (Fig. 2 c). Then, All the granulation tissue and dental stones in the extraction socket should be scraped and rinsed with 20ml normal saline. In control group the sockets only had blood clot; In test A group, autogenous bone, Bio-Oss (Geistlich, 0.25-1.00 mm, Switzerland) and barrier membrane (Z-H BIO, China, 20*15mm) [21] were applied in the extraction socket. First, autogenous bone was ground and mixed thoroughly with Bio-Oss, then the bone mixture was filled in the extraction socket till the height of the cemento-enamel junction; finally, the barrier membrane covered the surface of extraction socket. In test B group autogenous bone, Bio-Oss, CGF gel and CGF membrane were applied in the extraction socket. After centrifugation, two CGF test tubes were obtained. One was mixed with Bio-oss and autogenous bone thoroughly to form a gelatinous mixture for extraction socket filling. The other one was compressed into CGF membrane for extraction socket covering (Fig. 2. d-f). Two sutures were placed on the mesiodistal margins of the incision with 4 − 0 silk thread in all the three groups [22]. Postoperatively, amoxicillin (500 mg/8 h for five days), 0.2% chlorhexidine mouthwash (twice per day for seven days) and paracetamol (500 mg, every 4–6 h) were prescribed by the surgeon. All patients were given postoperative nursing guidance and sutures were removed at 7 days.

### Evaluation of outcomes

The investigator was not involved in the surgery and randomization, who was also not aware of the assignment of groups. Clinical data and imaging data were measured by two different investigators. The researchers analyzed the measurements of primary outcome variables including the height and width of bone repair in the extraction socket, and secondary outcome variables including PoSSe scale, PD and degree of gingival recession.

Postoperative symptom severity (PoSSe) scale was a questionnaire developed by Ruta et al. [23] to evaluate patients clinical symptoms after third molar extraction. A list of seven main adverse effects of extraction of molars was elicited including eating, speech, sensation, appearance, pain, sickness, interference with daily activities. The higher the score is, the more severe the symptom. The PoSSe scale will be filled by patient one week after extraction.

The PD of periodontal socket at distal buccal axis angle (PD-B), lingual axis angle (PD-L) and alveolar crest (PD-M) of the second molar were measured at 3rd, 6th and 12th months after surgery in all three groups. The distance (CG) between the cemento-enamel junction (CEJ) and the gum (G) of the second molar was measured to calculate the degree of gingival recession.

**Fig. 3:**
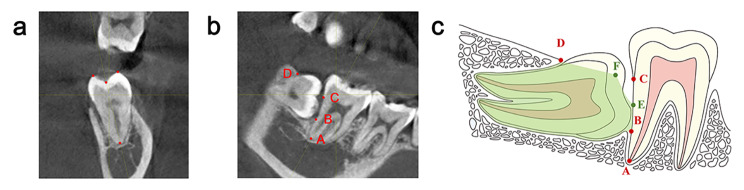
The CBCT measurement methods (**a**) sagittal plane, (**b**) coronal plane, (**c**) schematic diagram

Baseline values of each measurement index before extraction of the third molar were measured by CBCT: ① The distance between the distal root apex (point A) of the second molar and the lowest point (point B) where the third molar’s crown connects with the second molar, AB; ② The distance between point B and CEJ of the second molar (point C), BC; ③ The distance between the point C and the contact point (D) of the alveolar bone and the distal of the third molar, CD; ④ The lowest point (B) of the third molar connect with the distal root of the second molar is compared to the depth of the distal root of the second molar, AB/AC. In addition, the vertical height (BE) and horizontal distance (DF) of new bone at 3rd, 6th and 12th months after surgery, the value of ∠CEF, the relative vertical height (BE/BC) and relative horizontal distance (DF/CD) of new bone were measured (Fig. 3 a-c). The measurement methods and standards are as follows: on the CBCT graphics, the sagittal plane was determined by the three points of mandibular second molar: distal root apex, distal buccal tip and distal lingual tip of the crown. The coronal plane was determined by a plane which was vertical to the sagittal plane and cross both the central fossa point and the root apex point. Cross section images were captured at all time points, and superimposed together based on second molar anatomical markers (point A and C) for further measuring. All data were measured three times and averaged. By this method, the measurement plane could be relatively specific and constant, and also closer to the middle of the distal root of second molar, providing the measurements better accuracy and clinical meanings.

### Statistical analysis

The statistical analysis was performed by SPSS software (version 19.0, IBM, US), and the significance level was set at 5%. After normality test, the measurements were presented as averages and standard deviations. One-way ANOVA test was carried out to compare the difference among all three groups. For further analysis of the difference between the specific two groups, Least-Significant Difference test was performed. The bone repair at different time points in each group was analyzed by repeated measures ANOVA. For data do not follow a normal distribution, measurements were presented as medians (interquartile range), and nonparametric Kruskal-Wallis test was employed for post hoc analysis. In addition, Pearson correlation coefficient was used to depict possible relationship between bone defect and bone repair measurements.

## Results

A total of 75 patients were enrolled in this prospective randomized clinical trial from September 2020 to December 2021. 6 patients withdrew from the study without follow-up, 2 patients in the control group did not fully participate in the 12-month follow-up, and 1 patient in test B group did not fully participate in the study. Therefore, we ultimately enrolled 66 patients, including 35 females and 31 males, aged from 19 to 50 years old, with an average age of 29.32 ± 6.23 years old. There were 22 patients in control group, including 14 females and 7 males; 23 patients in test A group, 10 females and 13 males; 21 patients in test B group, 11 females and 10 males. None of the patients had the bone materials removal due to infection, 1 case of lower lip or lingual nerve numbness occurred in the control group, and 4 cases of nerve numbness occurred in test B group, all of them recovered spontaneously within 1 week.

At one week after surgery, there was no significant difference in PoSSe scales among all three groups. When the two test groups were compared separately, there were significant differences only in the time and degree of pain (p < 0.05), and no significant differences in the others (Table 1), indicating that the use of CGF could significantly alleviate short-term pain after tooth extraction.

**Table 1:** The PoSSe scale of all three groups

PoSSe score	Control (n = 22)	Test A (n = 23)	Test B (n = 21)	p value
	Mean ± SD (mm)	Mean ± SD (mm)	Mean ± SD (mm)	
Eating	12.77 ± 4.46	13.92 ± 5.68	11.50 ± 6.77	0.163
Speech	1.87 ± 1.96	0.90 ± 1.40	0.89 ± 1.13	0.984
Sensation	1.36 ± 3.57	0.00 ± 0.00	1.33 ± 2.48	0.081
Appearance	5.32 ± 2.57	4.89 ± 2.90	5.07 ± 3.44	0.842
Pain	7.88 ± 4.60	8.78 ± 4.60	5.88 ± 2.56	0.022*
Sickness	0.28 ± 0.94	0.00 ± 0.00	0.06 ± 0.27	0.728
Interference with daily activities	2.14 ± 2.25	2.56 ± 2.32	1.93 ± 2.43	0.703

The p value represents the statistical difference among the test groups (*Significant at the 5% level)

**Fig. 4:**
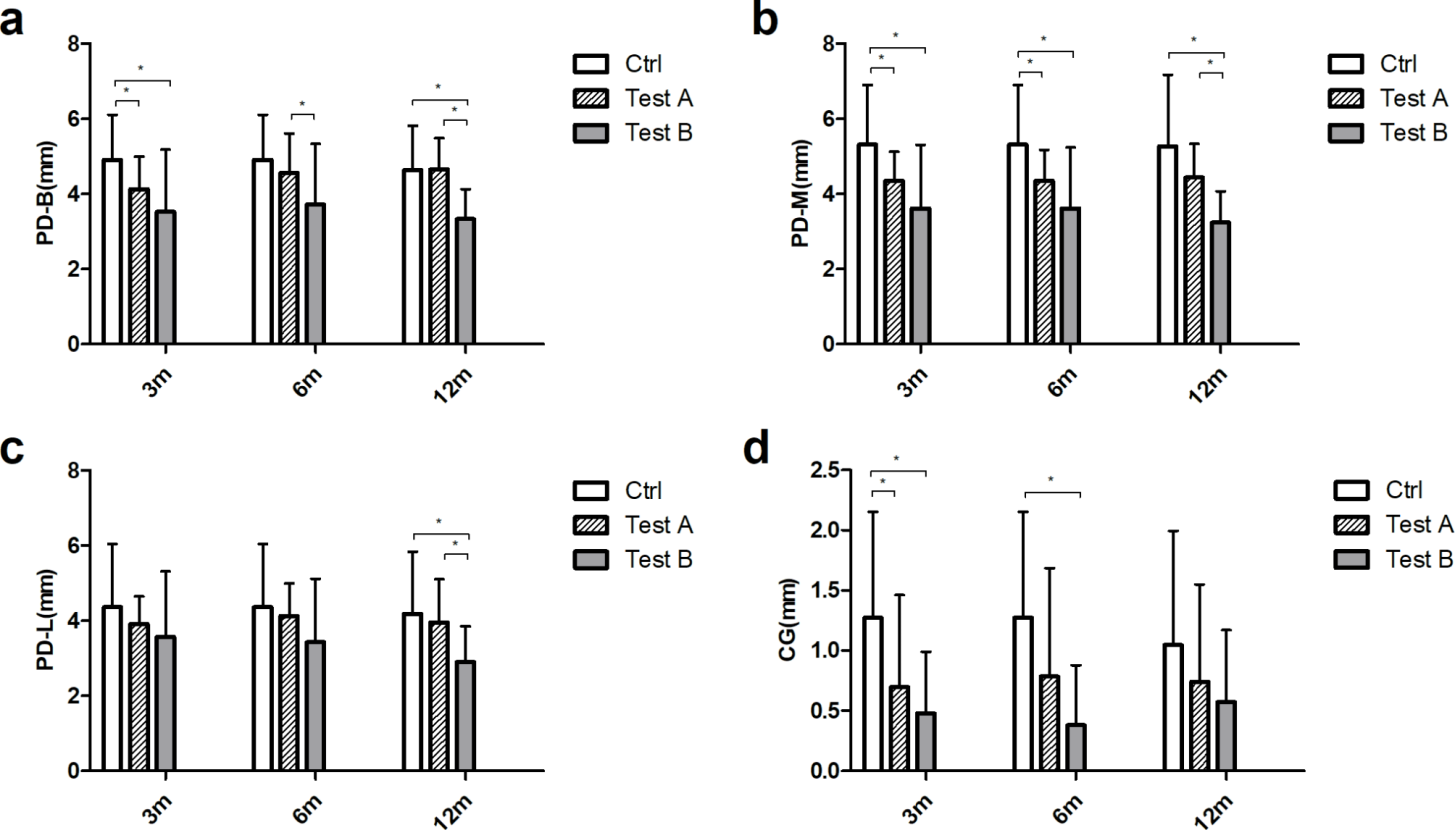
The periodontal probing depth and gingival recession measurements of all three groups at different time points. (**a**) PD-B, (**b**) PD-M, (**c**) PD-L, (**d**) CG. * represents p < 0.05

In terms of postoperative periodontal probing depth, the PD of the three groups at different time points were shown in Fig. 4 (Supplementary Table 1). At 3rd month, the PD-B, PD-M and CG values of the control group were significant different compared with those of test A group and test B group (P < 0.05), indicating that the two test groups both showed better periodontal repair. At 6th month, the difference between PD-B values of test A and test B group was statistically significant, while PD-M values of test A or test B group compared with control group were statistically significant, test B group exhibited more advantages (p < 0.05). At 12th month, the PD-B, PD-M and PD-L values of test B group were statistically significant compared with those of test A and control groups, and test B group was more advantageous (p < 0.05). At 12th month, there was no statistical difference in CG values among all three groups (Fig. 4).

**Fig. 5:**
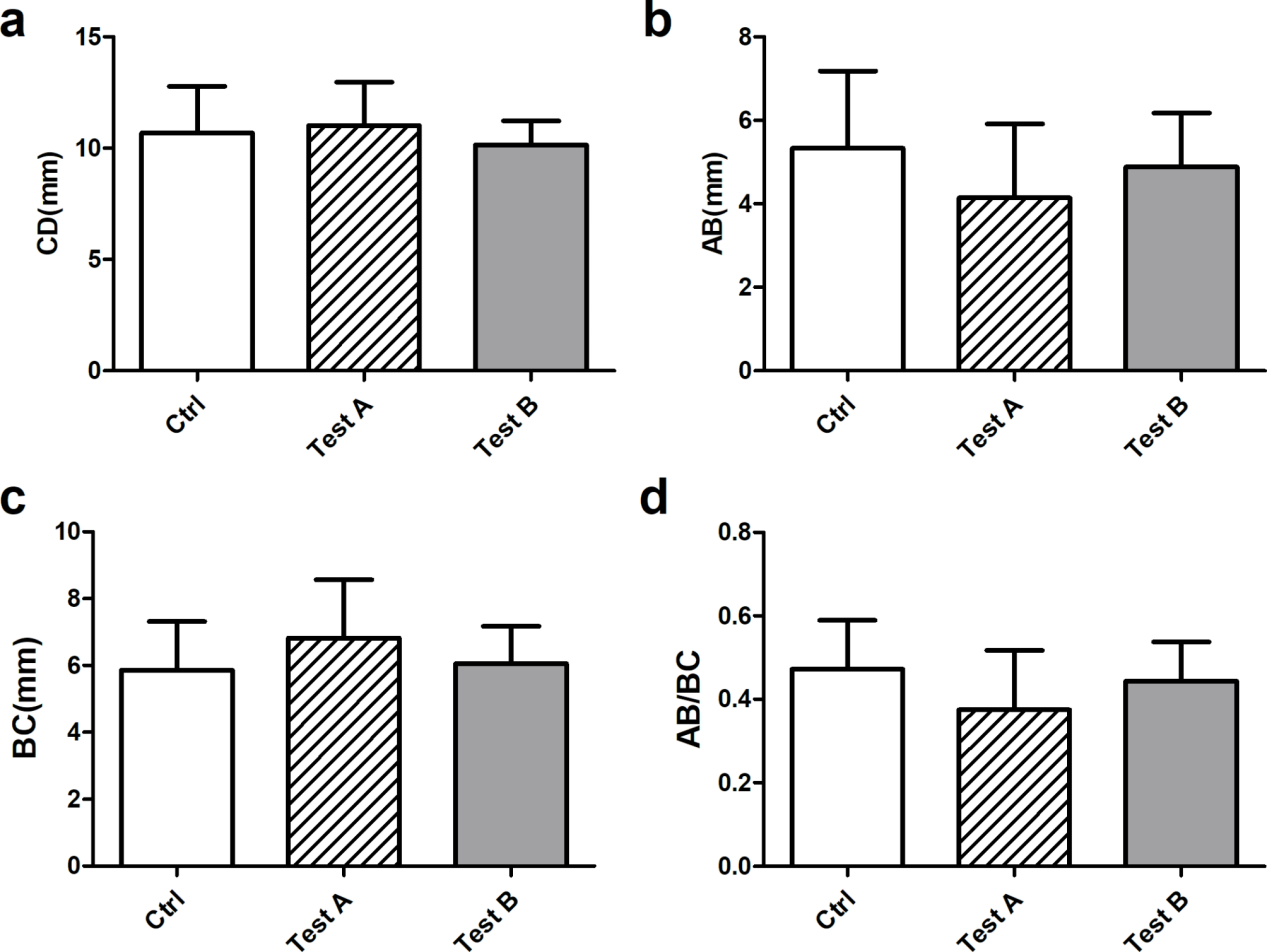
Baseline values before tooth extraction on the CBCT. (**a**) CD, (**b**) AB, (**c**) BC, (**d**) AB/BC. There was no statistical significance between three groups

**Fig. 6:**
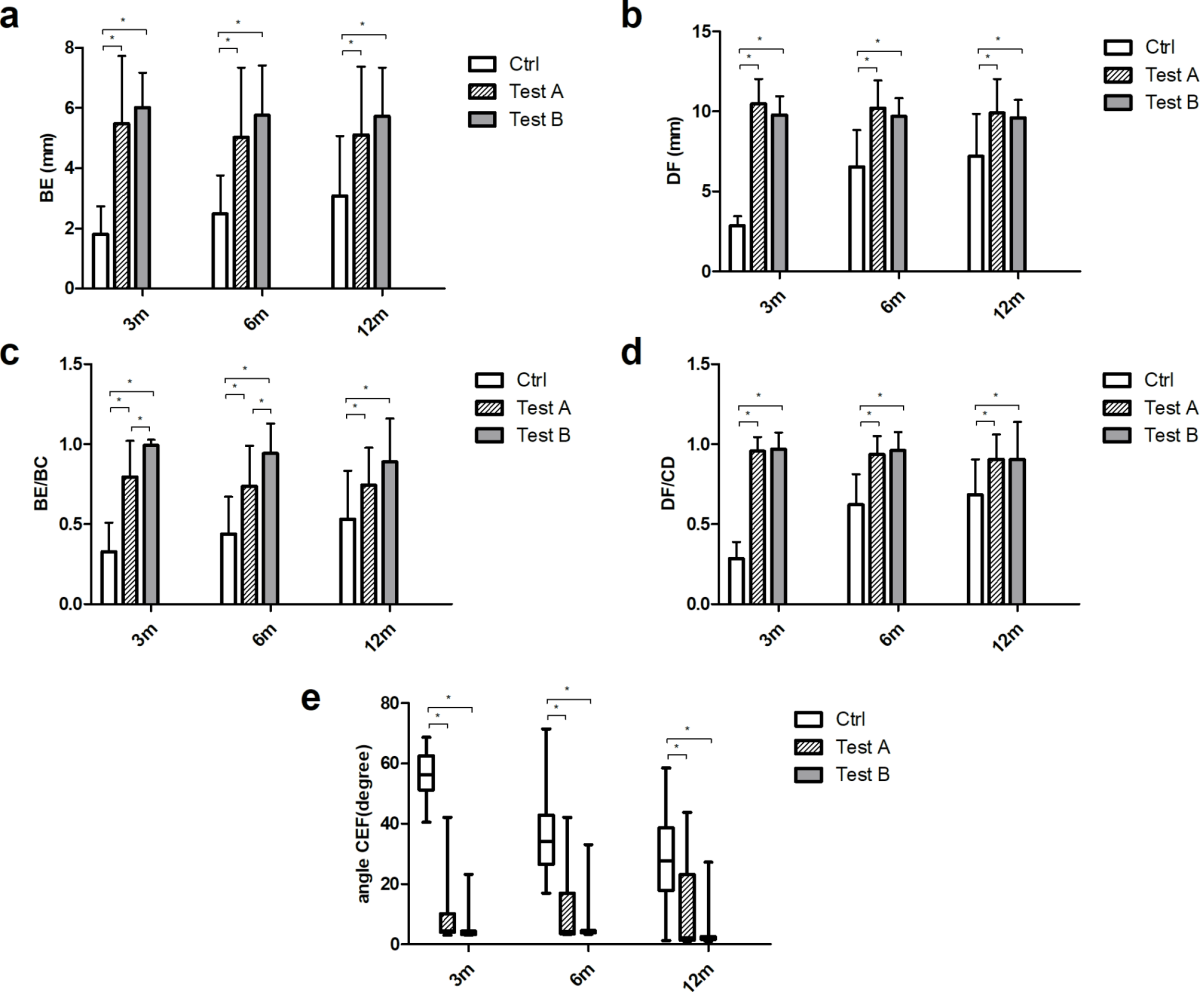
The CBCT measurements of all three groups at different time points. (**a**) BE, (**b**) DF, (**c**) BE/BC, (**d**) DF/CD, (**e**) ∠CEF, * represents p < 0.05

**Fig. 7:**
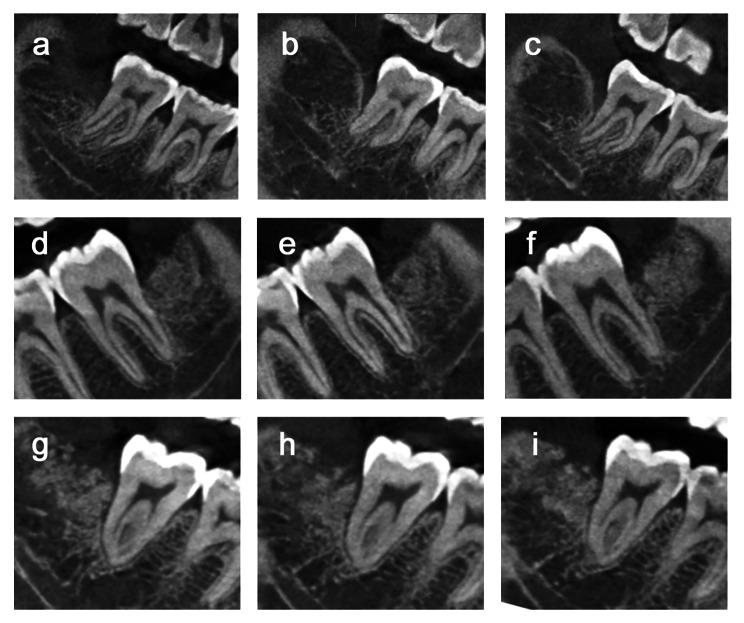
The CBCT images of all three groups at different time points. **(a-c)** 3rd, 6th and 12th month for control group; **(d-f)** 3rd, 6th and 12th month for Test A group; **(g-i)** 3rd, 6th and 12th month for Test B group

Regarding image data measured by CBCT, the baseline values of each measurements before tooth extraction were shown in Fig. 5 (Supplementary Table 2), and there was no significant difference among all three groups (Fig. 5 a-d).The measurement data of BE, DF, BE/BC, DF/CD and ∠CEF at different time points were shown in Fig. 5 (a ~ d: BE, DF, BE/BC and DF/CD were presented as mean and standard deviation; e: ∠CEF were presented as median and interquartile range; Supplementary Table 3). At all time, the BE, DF, BE/BC, DF/CD and ∠CEF of control group were statistically different compared with those measurements of test A and test B groups, indicating that both two test groups showed better bone repair results than control group (Table 2, P < 0.05). At 3rd and 6th month, the BE/BC values of test B group were significantly greater than test A (P < 0.05), yet there was no difference between the two groups at 12th month, indicating that CGF could promote the bone repair at early stage. At all points, there was no significant difference in BE, DF, DF/CD and ∠CEF between the two test groups (Fig. 6. a-e). CBCT images of all three groups at different time points were presented in Fig. 7, showing a better outcome of bone healing in both test groups (Fig. 7. D-F, G-I).

**Table 2:** The post-hoc analysis by least-significant difference test

Measurements	Time	Ctrl vs. Test A	Ctrl vs. Test B	Test A vs. Test B
PD-B	3rd month	0.062	0.001*	0.104
	6th month	0.377	0.004*	0.033*
	12th month	0.956	< 0.001*	< 0.001*
PD-M	3rd month	0.016*	0.001*	0.09
	6th month	0.022*	< 0.001*	0.086
	12th month	0.036*	< 0.001*	0.004*
PD-L	3rd month	0.334	0.062	0.343
	6th month	0.592	0.039*	0.114
	12th month	0.559	0.002*	0.009*
CG	3rd month	0.011*	0.001*	0.38
	6th month	0.041*	< 0.001*	0.097
	12th month	0.205	0.057	0.491
BE	3rd month	< 0.001*	< 0.001*	0.166
	6th month	< 0.001*	< 0.001*	0.183
	12th month	0.02*	0.01*	0.649
DF	3rd month	< 0.001*	< 0.001*	0.04*
	6th month	< 0.001*	< 0.001*	0.334
	12th month	< 0.001*	0.009*	0.302
BE/BC	3rd month	< 0.001*	< 0.001*	< 0.001*
	6th month	< 0.001*	< 0.001*	0.004*
	12th month	0.01*	< 0.001*	0.083
DF/CD	3rd month	< 0.001*	< 0.001*	0.664
	6th month	< 0.001*	< 0.001*	0.548
	12th month	0.001*	0.001*	0.999
∠CEF	3rd month	< 0.001*	< 0.001*	0.118
	6th month	< 0.001*	< 0.001*	0.452
	12th month	< 0.001*	< 0.001*	0.427

The p value represents the statistical difference between the test groups (*Significant at the 5% level)

According to Pearson correlation coefficient analysis, AB/AC and BE showed a significant negative correlation at 3rd, 6th and 12th month, indicating that the deeper position of third molar crown, the larger vertical height of new bone formation; At 12th month, there was a significant negative correlation between CD and BE, indicating that in the long run, the larger width of third molar exposure on bone surface, the smaller vertical height of new bone. There was no significant correlation between CD and DF/CD at different time points, indicating that there was no significant correlation between the horizontal distance of new bone and the meso-distal width of the third molar bone surface exposure (Table 3).

**Table 3:** Pearson correlation coefficient

	CD	AB/AC
AB/AC	0.172	1
BE 3rd month	-0.159	-0.349^**^
BE 6th month	-0.224	-0.305^*^
BE 12th month	-0.279^*^	-0.252^*^
DF/CD 3rd month	-1.91	
DF/CD 6th month	-2.41	
DF/CD 12th month	-1.66	

* P < 0.05, ** P < 0.01

## Discussion

At present, alveolar surgery usually experiences a process of “destruction before reconstruction”. Awareness of the clinical protection for the second molar was absent after third molar extraction. The operation process is often too casual, only focusing on how to remove the tooth. Such surgical procedures might result in excessive alveolar bone defects, leading to the risk of bone absorption in the distal area of the second molar, dentin sensitivity and even tooth extraction. In order to explore new strategies for the clinical protection of the second molar after third molar extraction, this study provided two different methods for the repair of the distal bone defect of the second molar. Both could provide effective clinical protection by reducing the formation of periodontal pocket, alleviating gingival recession, and prompting bone defect repair. Especially in terms of short-term pain reduction, early bone defect repair and long-term reduction of PD, test B group containing CGF exhibited more obvious advantages.

In terms of PoSSe scale, after tooth extraction there was almost no significant difference among all three groups except for pain perception. Test B containing CGF could significantly alleviate pain at the early stage, which was consistent with previous reports. Fang et al. [24] showed that the filling of CGF gel in tooth extraction could significantly reduce the pain responses 2 h, 24 and 48 h after surgery. Elayah et al. [25] also showed that CGF could significantly reduce pain caused by tooth extraction at 3 and 7 days after surgery. This might be attributed to the characteristics that a fibrous protein structure of CGF acted as a scaffold material and reservoir to transfer growth factors and helped accelerating the healing process [26]. In addition, Masuki et al. [27] detected increased levels of various growth factors in CGF, including PDGF, TGF-b1, VEGF and pro-inflammatory cytokines. These factors could significantly reduce pain caused by an inflammatory responses.

Concerning the depth of periodontal pocket and the degree of gingival recession, the PD-B and PD-M in test groups were significantly lower than that of the control group during short and long-term follow-up. Over 6 months, CGF and CGF membranes had better effects upon the healing of periodontal tissue and the reduction of gingival recession. CGF fibrin membrane is a blood agglutinate obtained by centrifuging venous blood specimens in a special centrifuge, which could produce fibrin with higher density and richer growth factors, therefore widely used in many clinical treatments and tissue engineering [28]. Li et al. [29] not only confirmed that CGF fibrin membrane could promote hUCMSCs-mediated periodontal tissue regeneration but also revealed that the promotion was achieved by upregulating the expressions of TAZ and genes related to osteogenic differentiation. Some scholars believed that the condition of periodontal recovery was also closely related to the age of patients at the time of tooth extraction, the degree of periodontitis, and the condition of bone defect [30]. Kugelberg et al. [31] found that without any treatment, distal periodontal bone defects of mandibular second molars in patients younger than 25 years old could be gradually recovered. However, in patients older than 25 years, distal periodontal bone defects of mandibular second molars were gradually aggravated [32].

Regarding defect repair, the vertical height and horizontal width of new bone of the two test groups were significantly better than that of the control group, and the angle of the distal infrabony pocket of the second molar was also significantly less than that of the control group. Bio-Oss has a porous structure as a bone substitute, which can guide the adhesion of osteoblasts, osteoclasts and so on, and change the structure of the filling material. It has been found that Bio-Oss can be retained for a long time in clinical application and participate in the process of new bone formation effectively [33]. Due to fast degradation rate of CGF in the oral cavity [34], biological scaffolds disappear prematurely in the process of alveolar bone regeneration and cannot maintain the osteogenic space for a long time, so it is better to mix them with other bone filling materials. In addition, the reuse of bone mass removed by ultrasonic osteotomy avoided the necessity of a second operative area for autogenous bone harvesting. Among them, cancellous structures such as cancellous bone or porous tissue engineering constructs have good osteogenic ability, which is conducive to the diffusion of nutrients and the reconstruction of tiny blood vessels [35, 36]. Although cortical bone has less bone induction and conduction ability, it can provide good mechanical support in the early stage of bone grafting, and its surviving osteoblasts can also provide certain osteogenic ability. While ultrasonic osteotomy can improve the clarity of visual field, reduce the probability of bone wall blood vessel bleeding and the risk of infection. When ultrasonic bone knife is used to cut bone tissue, the heat generated by high-focused ultrasound is less, and the use of condensed water can reduce the heat damage [37, 38]. Therefore, in view of the above aspects, we proposed to fill the extracted socket with the mixture of bone meal crushed by autogenous bone and Bio-Oss, CGF gel and CGF membrane. The process of alveolar bone remodeling, long-term maintenance of skeleton space and osteogenic ability all had good effects in our study.

The vertical height of new bone in test A and test B groups was significantly different at 3rd and 6th month, and the bone repair ability of test B group was significantly better than test A group, indicating that CGF gel and CGF membrane could significantly promote bone defect repair ability in the early stage, which was consistent with most previous reports [39, 40]. However, the long-term bone repair effect of two test groups was not significantly different. Our results might be attributed to the following two reasons: on one hand, because of the existing bone-guided materials, the promoting effect of CGF itself was less than that of combined intervention; on the other hand, the patients included in this study had a good recovery level without the influence of risk factors such as periodontitis, which could not be reflected in the present data. In clinical practise, since CGF is enriched from the patient’s autologous venous blood, it is convenient to obtain the materials, and involves no anticoagulants or chemical substances, cross-infection, immune rejection, or ethical controversy. The main device required is the variable-speed centrifuge. Compared with other biological materials, CGF is much safer, more effective, and more suitable for clinical application. The use of CGF gel and CGF membrane can reduce the use of Bio-Oss and barrier membrane, greatly relieve economic cost for patients. The active constituents of CGF could be affected by various factors including patient gender, age, as well as technical parameters of platelet concentrate preparation protocol. Francesco Bennardo et al. [41] innovatively combined PRF and antibiotics for drug delivery, providing the possibility of using autologous platelet concentrate as a natural carrier. Since the heterogeneity of CGF might impair its clinical efficacy, it is promising to combine CGF and certain ingredients such as growth factors for better clinical prognosticity in the future [41].

In addition, our study also found that in the control group, if the horizontal impacted crown of the third molar was exposed on the gum and contacted with the distal root of the second molar, the periodontal membrane might be completely lost, the vertical bone height in the extraction socket was difficult to recover, and the infrabony pocket was basically formed based on the lowest point of the crown of the third molar. If the crown of the third molar was not exposed, the vertical height and horizontal distance within the extraction socket could be well repaired. The unrepairable alveolar bone might be attributed to the loss of periodontal membrane in the distal root surface of the second molar, which was a result of the inflammatory factors induced by long-term exposure of the crown. The lower position of extracted third molar crown, the more obvious repair of the vertical bone; the larger meso-distal width of the exposed alveolar bone of third molar, the lesser repair of vertical bone. Due to the limited sample size, the bias of present measurement results might be amplified. If this result is to be confirmed, a larger sample size would be required for further investigation.

## Conclusion

The present study showed that in the clinical protection of mandibular second molar, both two test groups could achieve stable long-term bone defect repair. The use of CGF gel and CGF membrane could alleviate short-term pain after surgery, accelerate early-stage bone repair, reduce long-term probing depth and relieve economic cost for patients. Therefore, in the strategy of clinical protection of mandibular second molar, this new bone repair protocol is worthy of promotion by clinicians.

### Electronic supplementary material

Below is the link to the electronic supplementary material.

Supplementary Material 1

Supplementary Material 2

Supplementary Material 3

## Data Availability

The datasets used and/or analyzed during the study are available from the. corresponding author on reasonable request.

## Abbreviations

GTR/GBR: Guided tissue/bone regeneration
PRF: Platelet rich fibrin
DDM: Demineralized dentin matrix
CGF: Concentrate growth factors
PRP: Platelet-rich plasma
PD: Probing depth
PoSSe: Post operative symptom severity
CEJ: Cemento-enamel junction
